# Human Serine Racemase Weakly Binds the Third PDZ Domain of PSD-95

**DOI:** 10.3390/ijms23094959

**Published:** 2022-04-29

**Authors:** Roberta Giaccari, Francesco Marchesani, Carlotta Compari, Emilia Fisicaro, Andrea Mozzarelli, Barbara Campanini, Stefano Bettati, Stefano Bruno, Serena Faggiano

**Affiliations:** 1 Department of Food and Drug, University of Parma, 43124 Parma, Italy; roberta.giaccari@studenti.unipr.it (R.G.); francesco.marchesani@unipr.it (F.M.); carlotta.compari@unipr.it (C.C.); emilia.fisicaro@unipr.it (E.F.); andrea.mozzarelli@unipr.it (A.M.); barbara.campanini@unipr.it (B.C.); stefano.bruno@unipr.it (S.B.); 2 Institute of Biophysics, CNR, 56124 Pisa, Italy; stefano.bettati@unipr.it; 3 Biopharmanet TEC, University of Parma, 43124 Parma, Italy; 4 Department of Medicine and Surgery, University of Parma, 43124 Parma, Italy

**Keywords:** serine racemase, D-serine, PDZ domain, PSD-95, SAP-90, DLG4, protein–protein interaction, N-methyl-D-aspartate receptor

## Abstract

Human serine racemase (hSR) is a pyridoxal-5′-phosphate (PLP)-dependent dimer that catalyzes the formation of D-serine from L-serine, as well as the dehydration of both L- and D-serine to pyruvate and ammonia. As D-serine is a co-agonist of N-methyl-D-aspartate receptors (NMDARs), hSR is a key enzyme in glutamatergic neurotransmission. hSR activity is finely regulated by Mg^2+^, ATP, post-translational modifications, and the interaction with protein partners. In particular, the C-terminus of murine SR binds the third PDZ domain (PDZ3) of postsynaptic density protein 95 (PSD-95), a member of the membrane-associated guanylate kinase (MAGUK) family involved in the trafficking and localization of glutamate receptors. The structural details of the interaction and the stability of the complex have not been elucidated yet. We evaluated the binding of recombinant human PSD-95 PDZ3 to hSR by glutaraldehyde cross-linking, pull-down assays, isothermal titration calorimetry, nuclear magnetic resonance, and enzymatic assays. Overall, a weak interaction was observed, confirming the binding for the human orthologs but supporting the hypothesis that a third protein partner (i.e., stargazin) is required for the regulation of hSR activity by PSD-95 and to stabilize their interaction.

## 1. Introduction

Mammalian serine racemase (SR) (EC:5.1.1.18) is a homodimeric pyridoxal-5′-phosphate (PLP)-dependent enzyme responsible for the biosynthesis of D-serine in the brain, thus representing a crucial enzyme for the modulation of the glutamatergic neurotransmission mediated by N-methyl-D-aspartate receptors (NMDARs), of which D-serine is a co-agonist [1,2,3,4,5,6]. D-serine is both produced and degraded by SR: the racemization of L-serine forms D-serine, while both L-serine and D-serine are transformed into pyruvate and ammonia by β-elimination [7]. The alteration of D-serine levels in the central nervous system (CNS) has been linked to several neuropathologies, such as Alzheimer’s disease, Parkinson’s disease, and schizophrenia [8]. Although a significant effort has been devoted to identifying small molecules able to modulate the activity of human serine racemase (hSR), only low-affinity inhibitors targeting the active site have been developed so far, pointing to the need for a different strategy to discover drug therapeutics that modulate D-serine levels [9,10,11].

The activity of SR is regulated by several effectors, including metal ions, small molecules, and proteins [2,12,13,14,15,16,17]. Among its protein ligands, SR has been reported to bind proteins containing PDZ domains, such as GRIP, PICK1, DISC1, and PSD-95 [18,19,20,21], which interact with the C-terminus of their protein targets. PDZ domains were originally classified into three groups according to the last three residues at the C-terminus of their peptide ligands: Class I PDZ domains bind to C terminal motifs with the sequence Ser/Thr-X-Φ-COOH, Class II PDZs to the sequence Φ-X-Φ-COOH, and Class III PDZs to the sequence Asp/Glu-X-Φ-COOH (Φ is any hydrophobic amino acid and X is any amino acid) [22,23]. However, binding promiscuity has emerged more recently as a distinguishing feature of PDZ domains [24,25]. The interaction between PDZ domains and consensus C-terminal sequences is mainly driven by hydrophobic interactions and hydrogen bonds formed in the carboxylate binding groove [26].

Postsynaptic density protein 95 (PSD-95, also known as SAP-90 or DLG4), a membrane-associated guanylate kinase (MAGUK) involved in the trafficking and localization of α-amino-3-hydroxy-5-methyl-4-isoxazolepropionic acid (AMPA) and NMDA glutamate receptors [27], has been reported to interact with murine SR (mSR) [28]. The interaction with PSD-95 was at first identified in HEK-293 cell lysates by co-immunoprecipitation of mSR and a library of GST-tagged human proteins. Further mapping experiments using different truncated constructs of PSD-95 allowed to determine that the portion of PSD-95 responsible for the binding was its third PDZ domain (PSD-95 PDZ3) [28]. Deletion of the last four amino acids in mSR abolished binding, proving that the interaction occurred via the PDZ consensus sequence (-TVSV) present at mSR C-terminus. It was also suggested that murine SR, PSD-95, and stargazin, an AMPA receptors (AMPAR) accessory protein, form a ternary complex in which the activity of SR is reduced, and which is involved both in the regulation of glutamatergic neurotransmission and in the cross-talk between AMPAR and NMDAR [28]. Further studies indicated that hSR is associated with PSD-95 and NMDARs in human postsynaptic neurons and that this interaction modulates glutamatergic synaptic development, suggesting that it interacts with PSD-95 PDZ3 as its murine ortholog [29].

In this work, we evaluated the binding of hSR and human PSD-95 PDZ3 by exploiting different biochemical and biophysical techniques.

## 2. Results

### 2.1. Pull-Down Assays exclude the Presence of a Strong Binding between hSR and PSD-95 PDZ3

**Figure 1:**
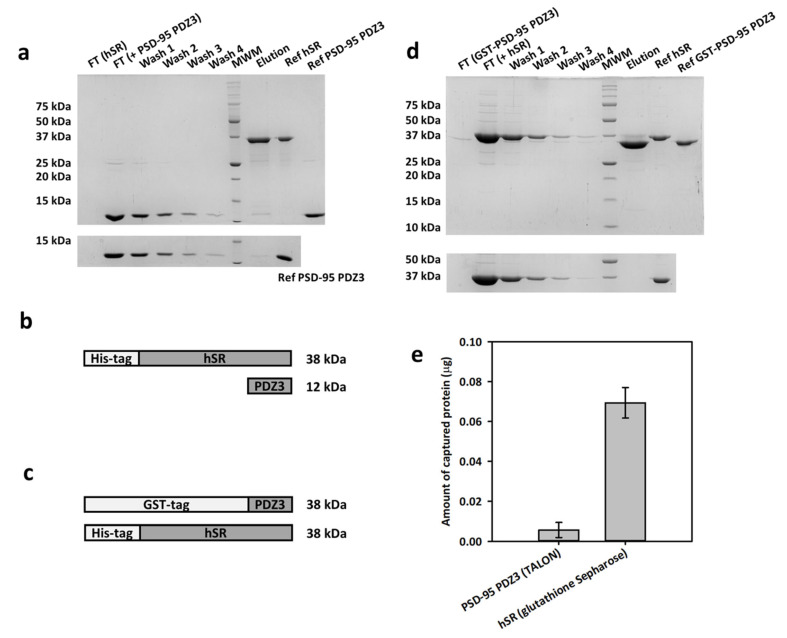
Pull-down assays by immobilizing either hSR or GST-PSD-95. (**a**) (**Top**) pull-down assay on TALON^®^ resin, immobilizing His-tagged hSR. FT is the flow-through after protein equilibration; (**bottom**) control experiment with only PSD-95 PDZ3 loaded onto the resin. (**b**) Scheme of the proteins used for the pull-down experiments with TALON^®^ resin. (**c**) Scheme of the proteins used for the pull-down experiments with glutathione Sepharose^®^ resin. (**d**) (**Top**) pull-down assay on glutathione Sepharose^®^ resin, immobilizing GST-PSD-95 PDZ3; (**bottom**) control experiment with only hSR loaded. (**e**) Amount of PSD-95 PDZ3 (**left**) and hSR (**right**) specifically captured by immobilized hSR and GST-PSD-95 PDZ3, respectively, obtained by the densitometric analysis of the band intensities for each condition. The pull-down experiments were performed in triplicate and the standard error of the densitometric analysis is reported.

To assess if hSR, which has a C-terminal PDZ consensus sequence (-SVSV) similar to mSR (Supplementary Materials, Figure S1), forms a stable complex with PSD-95 PDZ3, we carried out pull-down assays in which we immobilized one of the two proteins onto an affinity resin (IMAC and glutathione Sepharose^®^ for hSR and GST-PSD-95 PDZ3, respectively) and evaluated the co-immobilization of the second protein (Figure 1). Pull-down assays typically identify interacting proteins only if the complex is sufficiently stable; hence, this technique was applied to obtain a preliminary indication of binding [30]. At first, we immobilized hSR on a TALON^®^ resin by exploiting its His-tag, and evaluated the co-immobilization of PSD-95 PDZ3 (Figure 1a, top, scheme in Figure 1b). As a control, we performed the same experiment in the absence of hSR to assess the non-specific binding of PSD-95 PDZ3 to the resin (Figure 1a, bottom). Upon elution with imidazole, a faint band corresponding to PSD-95 PDZ3 was observed in the eluate, with lower intensity compared to the band corresponding to the last wash step (Figure 1a, top). A more extensive resin wash (400 µL instead of 200 µL) resulted in the total absence of the band related to PSD-95 PDZ3 in the last wash step and in the elution phase, confirming that no binding to hSR occurs under these conditions (Supplementary Information, Figure S2). The experiment was repeated using a Ni^++^ NTA resin to check if the IMAC resin might affect the result. However, non-specific binding of PSD-95 PDZ3 to the resin was observed, thus preventing the detection of the complex (Supplementary Materials, Figure S3).

An alternative experiment was performed by exploiting a complementary immobilization scheme (Figure 1c,d). Although GST-PSD-95 PDZ3 and hSR have a very similar molecular weight (MW), their electrophoretic mobility differs enough to distinguish the two proteins in SDS-PAGE. The GST-PSD-95 PDZ3 domain was, therefore, immobilized onto a glutathione Sepharose^®^ resin and incubated with hSR (Figure 1d, top). Upon elution with glutathione, a band corresponding to hSR was observed in the eluate, with an intensity slightly higher compared to the last wash step (Wash 4), suggesting that a very small amount of hSR was bound to immobilized GST-PSD-95 PDZ3 (Figure 1d). A control experiment excluded non-specific binding of hSR to the glutathione Sepharose^®^ resin (Figure 1d, bottom).

We performed a densitometric analysis of the band intensities in the elution lanes for both experiments and controls. The amount of PSD-95 PDZ3 captured by specific binding to hSR immobilized on the TALON^®^ resin is close to zero, indicating that no binding occurred between the two proteins in this set of experiments (Figure 1e, left). On the other hand, the amount of hSR captured by specific binding to GST-PSD-95 PDZ3 immobilized on glutathione Sepharose^®^ resin is small but significantly different from zero, confirming that binding between the two proteins occurs, although to a very low extent (Figure 1e, right).

Overall, the pull-down assays indicated the absence of strong binding between the two proteins.

### 2.2. NMR Titration Confirms That the Binding between the Two Proteins Is Weak

**Figure 2:**
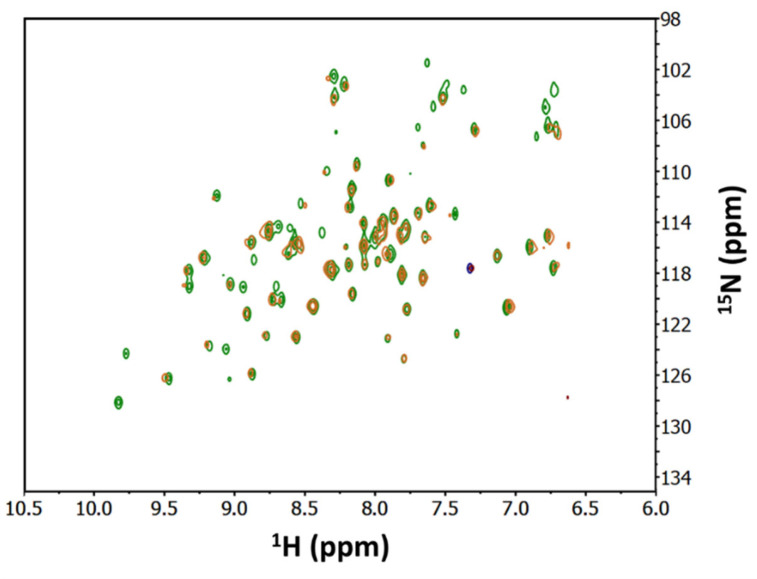
^1^H-^15^N HSQC NMR spectra of 80 µM ^15^N-labeled PSD-95 PDZ3 alone (green) and upon addition of 1:3 hSR (orange). Only the initial (no hSR) and final (1:3 PDZ3:hSR) spectra of the titration are reported.

Nuclear magnetic resonance can be exploited to assess protein–protein interactions and has been applied to the characterization of protein complexes involving PDZ domains [31,32]. Chemical shift perturbation (CSP) analysis is the most widely applied NMR method for the determination of dissociation constants in the μM-mM range with exchange regime between the free and ligated form defined as “fast” on the NMR timescale (exchange rate ≥ μs^−1^) [33,34]. To stabilize the complex, we performed NMR titration experiments at higher protein concentrations (80 μM for ^15^N-labeled PSD-95 PDZ3 and up to 240 μM for hSR) compared to the pull-down assays (Figure 2). A ^1^H-^15^N HSQC spectrum of ^15^N labeled PSD-95 PDZ3 was first acquired and showed a good dispersion of the peaks, indicating proper protein folding. hSR was then added up to a 1:3 molar ratio. It was not possible to further increase the hSR/PDZ3 ratio due to hSR tendency to aggregate at higher concentrations.

No significant chemical shift perturbation of the peaks was observed upon the addition of 1:3 hSR, suggesting that no interaction occurs. Another possible effect of the interaction, due to the relatively high MW of hSR dimer (76 kDa), is the disappearance of PDZ3 spectrum due to the much higher MW of the complex compared to PSD-95 PDZ3 (12 kDa). In fact, the S/N ratio of an HSQC spectrum is strongly reduced if the tumbling rate of the macromolecule in solution is slow, due to a high MW. In the hypothesis that one dimer of hSR binds two PDZ3 domains, the MW of the complex would be about 100 kDa. Upon addition of 1:3 hSR, only a weak reduction of the intensity of the spectrum was noted, which might possibly be linked to the slight dilution of PSD-95 PDZ3 during the experiment.

Overall, the NMR experiments suggested that hSR and PSD-95 PDZ3 either do not interact, or interact with low affinity, and the complex is, therefore, not detectable at the concentrations used for these measurements.

### 2.3. Cross-Linking Experiments Suggest That hSR Does Bind to PSD-95 PDZ3

**Figure 3:**
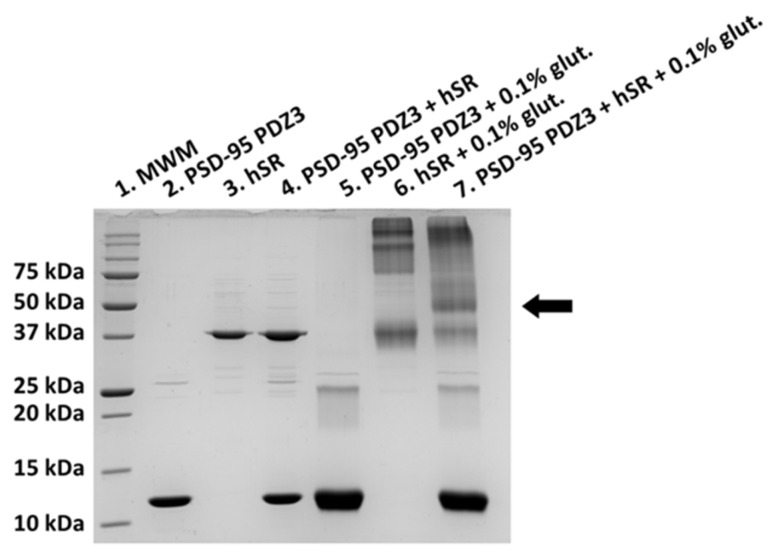
SDS-PAGE after cross-linking with 0.1% glutaraldehyde of hSR and PSD-95 PDZ3. Lane 1: MW marker. Lanes 2, 3, and 4: reference proteins with no glutaraldehyde; lane 2: PSD-95 PDZ3, lane 3: hSR, and lane 4: PSD-95 PDZ3 plus hSR. Lanes 5, 6, and 7: proteins with 0.1% glutaraldehyde; lane 5: PSD-95 PDZ3, lane 6: hSR, and lane 7: PSD-95 PDZ3 plus hSR. An arrow indicates the band at 50 kDa.

After NMR and pull-down experiments ruled out the presence of a stable complex between hSR and PSD-95 PDZ3, we carried out chemical cross-linking experiments to check for the formation of weak protein complexes [35]. Upon incubation of glutaraldehyde (0.1% *w*/*v*) with a mixture of hSR (15 μM) and PSD-95 PDZ3 (30 μM), we observed in SDS-PAGE a new species with a MW of 50 kDa, equal to the sum of the MWs of hSR (monomer: 38 kDa) and PSD-95 PDZ3 (12 kDa), suggesting that the two proteins interact in solution (Figure 3). The band was not present in the control experiments in which only hSR or PSD-95 were reacted with glutaraldehyde, confirming that only the simultaneous presence of both proteins permits the formation of the 50 kDa species. In the control experiments with hSR only, high MW conjugates were observed, as expected since hSR is a dimer and the subunits are, therefore, likely to react. In those with PSD-95 PDZ3 only, a faint band corresponding to a dimeric adduct at 25 kDa was observed, which also formed when PSD-95 PDZ3 and hSR were reacted. However, the band at 50 kDa corresponding to the hSR-PDZ3 adduct was six-times more intense by densitometric analysis than that at 25 kDa, confirming the formation of a transient complex between hSR and PDZ3 and ruling out a non-specific reaction.

The fact that the interaction was detected by cross-linking, but not by pull-down or NMR, suggests that the two proteins do interact but form a weak complex in vitro.

### 2.4. ITC Titrations Confirm That hSR Binds with Low Affinity to PSD-95 PDZ3

We applied isothermal calorimetry titration to retrieve thermodynamic information about the binding between the two proteins. ITC has been applied to determine binding affinities for various PDZ domains and peptides, measuring K_D_ values spanning from the high-nanomolar to the high-micromolar range [36,37,38].

**Figure 4:**
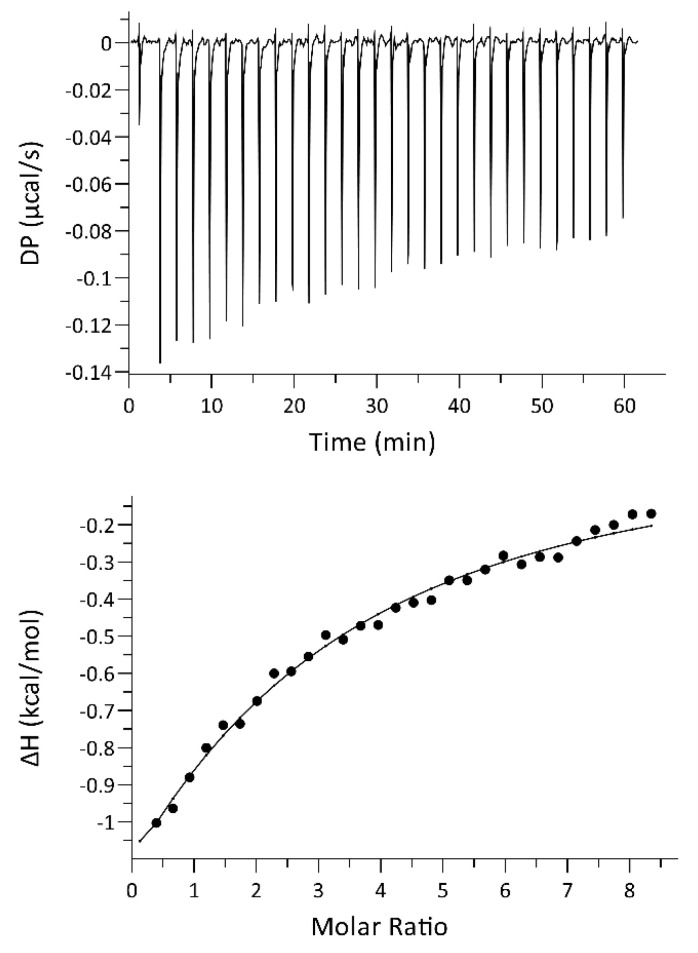
ITC experiment for hSR (20 µM) titration with PSD-95 PDZ3 (600 µM). Raw data for ITC titration (**top**) and binding isotherm of the integrated titration curve (**bottom**) were obtained at 25 °C in 50 mM HEPES buffer, pH 8.0.

We performed several experiments by varying the concentration of both proteins. In all experiments, an exothermic reaction was observed, with very low heat, close to the limit of detection of the instrument. The binding curve and the relative binding isotherm is reported in Figure 4. The fitting of the curve using a single-site model yielded a dissociation constant of 82 ± 14 µM.

### 2.5. The Enzymatic Activity of hSR Is Moderately Increased by the Presence of PSD-95 PDZ3

**Figure 5:**
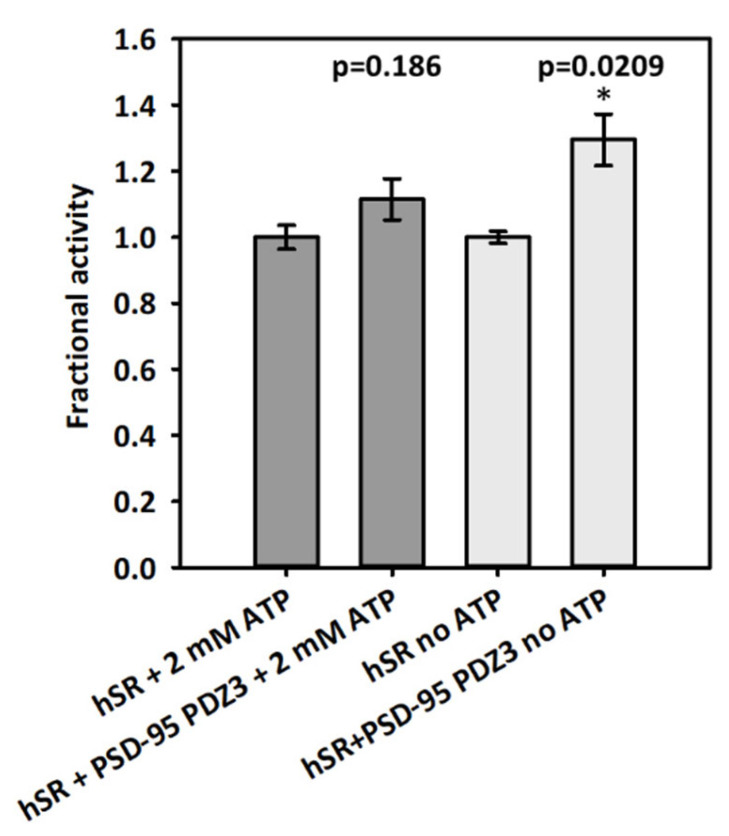
Effect of PSD-95 PDZ3 on hSR activity, in the presence (dark grey bars) and absence (light grey bars) of ATP. The fractional activity is reported setting as 1 the activity without PSD-95 PDZ3 either with 2 mM ATP or without ATP. All experiments were performed in triplicate. The *p* values from the statistical analysis of the sets of experiments with or without ATP are reported on the graph. The symbol * indicates a statistically significant difference between the two fractional activities with or without PSD-95 PDZ3 in the absence of ATP.

We tested the enzyme activity of hSR (0.44 µM) on the β-elimination of L-serine in the absence and presence of PSD-95 PDZ3 at 500 μM, the highest concentration that could be reached in the assay mixture (Figure 5). Under these conditions, considering the K_D_ of 82 µM obtained in ITC experiments, about 85% of hSR would be in complex with PDZ3. The effect of PDZ3 on hSR activity was measured both in the presence and absence of ATP, an allosteric effector known to increase hSR activity by promoting the stabilization of a more active conformation [15,16]. hSR activity increased by 22% when PSD-95 PDZ3 was added to the assay in the absence of ATP, whereas in the presence of ATP the activity did not significantly change (Figure 5).

## 3. Discussion

The regulation of hSR activity by protein interactors is still obscure in many respects, particularly regarding its molecular aspects. These details can only be unveiled by an in vitro characterization using isolated proteins, in order to dissect the pieces of evidence revealed by cellular studies.

The characterization of the interaction between hSR and PSD-95 PDZ3, carried out by applying complementary biochemical and biophysical techniques, pointed to a very weak binding. This result can be compared to published results on murine SR [28]. Al-though no K_D_ value was reported for its interaction with PSD-95 PDZ3, a hypothetical range of affinities can be retrieved considering that it was identified by co-immunoprecipitation, which detects only stable complexes (expected K_D_s are in the nano- or low micro-molar range). This suggests that the murine enzyme has a much higher affinity for PSD-95 PDZ3 compared to hSR in this study.

The effect of the presence of PSD-95 PDZ3 on hSR enzyme activity is similar to what was reported for the murine ortholog, which was not significantly inhibited by PSD-95 in cell lysates [28], in conditions corresponding to our experiments in the presence of ATP. We were also able to assess a slight increase of the activity in the presence of PSD-95 PDZ3 and absence of ATP, a further indication of direct interaction.

In the binding of C-terminal moieties of proteins to PDZ domains, positions 0 (the last amino acid) and -2 (the third last amino acid) are considered the most important to determine binding specificity [22,39], although also position -3 (the fourth last amino acid) is in direct contact to the peptide-binding groove and has a role in the binding; furthermore, residues beyond the last 4 amino acids up to position -8 are involved in the interaction. Specific studies proved the importance of at least 8 amino acids of a binding peptide for the interaction with PSD-95 PDZ3 [40]. It was reported that the interaction of PDZ3 with the C-terminal peptide of cysteine-rich PDZ-binding protein (CRIPT), one of its natural ligands, terminating as -YKQTSV, involves the canonical carboxylate binding groove as well as a C-terminal helix (α3) which forms part of an extra binding surface [41]. Another study of interaction with the hexapeptide KKETAV indicated that the lysine in position -4 is critical, being involved in ionic interactions with Glu331, Asp332, and Glu334 residues in PSD-95-PDZ3 [42]. Notably, PSD-95 PDZ3 is classified as a class I PDZ domain (consensus sequence Ser/Thr-X-Φ-COOH) [43] and most interaction studies have been performed with peptides having a threonine at position -2. However, the interaction with mSR, having a hydrophobic valine residue in position -2, pointed to a promiscuous behavior of PSD-95 PDZ3 [28].

The last eight residues of hSR and mSR sequences are -PASYQSVSV and -PAPYQTVSV, respectively (Supplementary Information, Figure S1). Two residues are different, in position -3 (S in hSR, T in mSR) and -6 (S in hSR, P in mSR). In particular, the presence of a proline in position -6 may provide increased rigidity to the C-terminal moiety of the murine ortholog, possibly favoring the interaction of mSR with PSD-95 PDZ3. Therefore, it is possible that these changes are responsible for the different binding affinities observed for the two orthologs. The amino acid sequence of PDZ3 PSD-95 is identical for human and murine orthologs, so this is not a factor affecting the observed differences. The last 10 amino acids are not resolved in any hSR X-ray structure deposited in the Protein Data Bank, indicating that the C-terminus is highly flexible and potentially extends out of the bulk surface of the protein, thus allowing the interaction with the PDZ domain.

The transient interaction between human SR and PSD-95 might be functional to a regulatory role, allowing a tunable activity modulation upon interaction with PSD-95 in neurons. Indeed, the presence of highly dynamic interactions between PDZ domains and their binding partners has already been demonstrated [36,44,45,46]. Weak affinities assist the physiological modulation of the function of the target protein, as in the case of the cystic fibrosis transmembrane conductance regulator (CFTR) [47]. Moreover, hSR post-translational modifications, such as S-nitrosylation at C113 of hSR, could affect binding to PSD-95, since the cysteine lays on the same side of the protein surface as the the C-terminus [48,49,50]. Phosphorylation of PSD-95 PDZ3 might as well modulate the binding [51].

Finally, it should be considered that other proteins, such as the neuronal AMPAR-associated membrane protein stargazin, could be part of the scenario and stabilize the ternary complex with hSR and PSD-95 binding its second PDZ domain, similarly to what reported for murine SR [28]. Experiments on cell lysates demonstrated that, for murine SR, the formation of a ternary complex comprising SR, PSD-95, and stargazin causes a reduction of SR activity, associated to SR recruitment in close proximity to cell membrane [28]. It is therefore likely that this mechanism is also in play for hSR, for which the colocalization with PSD-95 in post-synaptic neurons has been proved [29]. Allostery of PSD-95 PDZ domains might also play a role in modulating the binding affinities of protein partners [52,53].

Further investigations are needed to decipher the molecular details of hSR/PSD-95 interaction and the role of other protein partners in modulating the complex formation and hSR enzymatic activity.

## 4. Materials and Methods

### 4.1. Chemicals

Chemicals were of the best commercial quality available and were purchased from Merck-Millipore (Darmstadt, Germany), unless otherwise stated.

### 4.2. Purification of hSR and PSD-95 PDZ3

Recombinant hSR was expressed as a hexa-His tagged fusion protein encoded in a pET28a-derived plasmid [54] transformed into *E. coli* BL21 CodonPlus^®^ (DE3)-RIL cells, previously transformed with plasmids encoding GroEL and GroES chaperonins. These chaperonins were selected from the Chaperone Plasmid set, Takara^®^, as they increased the expression of soluble hSR [55]. The His-tag is present at the N-terminus of the protein. Expression and purification on a TALON^®^ His-tag purification resin (Clontech, Mountain View, CA, USA) were performed as reported elsewhere [55]. hSR was equilibrated in 50 mM N-(2-hydroxyethyl) piperazine-N’-ethanesulfonic acid (HEPES) pH 8.0 for pull-down assays, cross-linking, ITC titrations, and enzymatic assays and in 50 mM Na phosphate pH 7.8 for NMR experiments. Protein purity was assessed as 88% by densitometry of Coomassie Blue-stained bands of an SDS-PAGE gel using a ChemiDoc gel imaging system (Bio-Rad, Hercules, CA, USA). All concentrations for hSR are expressed as a monomer.

PSD-95 PDZ3 was expressed as a GST-tagged fusion protein encoded in a pGEX-6P-1 plasmid transformed into *E. coli* BL21 CodonPlus^®^ (DE3)-RIL cells. The plasmid was a gift from Sachdev Sidhu (Addgene plasmid #103953; http://n2t.net/addgene:103953, accessed on 1 February 2019; RRID:Addgene_103953) [23]. Cells were grown at 37 °C in Luria Bertani medium supplemented with 100 μg/mL ampicillin. When the cells reached an OD at 600 nm of ~0.6, isopropyl β-D-1-thiogalactopyranoside (IPTG) was added to a final concentration of 1 mM and the culture was further incubated at 37 °C for 4 h.

For NMR experiments, ^15^N labeled PSD-95 PDZ3 was expressed using M9 minimal medium added of 1 g/l ^15^N ammonium sulfate (Cambridge Isotopes) as the sole source of nitrogen. The ^15^N labeled PSD-95 PDZ3 was expressed similarly to the unlabeled protein. Cells were harvested by centrifugation and the pellet was resuspended in phosphate-buffered saline (PBS) pH 7.4 added with 5 mM 1,4-dithiothreitol (DTT), 0.2 mM phenylmethylsulfonyl fluoride (PMSF), 0.2 mM benzamidine, and 1.5 μM pepstatin. Cells were disrupted by treatment with lysozyme (1 mg/mL), followed by sonication. The homogenate was clarified by centrifugation (16,000× *g*, 50 min, 4 °C) and the supernatant was loaded onto a glutathione Sepharose^®^ 4B column (Cytiva). The fusion protein was eluted with 50 mM tris(hydroxymethyl)aminomethane (Tris), 5 mM 1,4-dithiothreitol (DTT), 10 mM reduced glutathione pH 8.0. GST tag cleavage was performed by incubation with PreScission™ Protease (Cytiva) in 50 mM Tris, 100 mM NaCl, 1 mM EDTA, 5 mM DTT pH 7.4 O/N at 4 °C. The solution was loaded again on the glutathione Sepharose^®^ 4B column to separate the PDZ3 domain from cleaved GST. Buffers used for the final equilibration of PSD-95 PDZ3 are the same mentioned before for hSR. Protein purity was assessed as 96%.

### 4.3. Pull-Down Assays

The pull-down assays were performed in 50 mM HEPES buffer, pH 8.0 using 50 μL of TALON^®^ His-tag purification resin (Clontech, Mountain View, CA, USA) or glutathione Sepharose^®^ as affinity support.

The TALON^®^ resin was incubated for 1 h at 4 °C with 30 μM hSR. The flow-through was collected and the resin was incubated with 30 μM PSD-95 PDZ3 for 30 min at 4 °C. The flow-through was collected and the resin was washed with a total of 200 μL of 50 mM HEPES pH 8.0 divided in four fractions of 50 μL. hSR was then eluted using 200 μL of 50 mM Na phosphate, 150 mM NaCl, 250 mM imidazole pH 8.0 divided in four fractions of 50 μL. Protein fractions were analyzed by SDS-PAGE. A similar experiment was performed with the glutathione Sepharose^®^ resin, incubating GST-PSD-95 PDZ3 at first, and hSR in the second step. The resin was washed with 200 μL of 50 mM HEPES pH 8.0 in 4 separate steps of 50 μL each and the bound PSD-95 PDZ3 was eluted in 200 μL of 50 mM Tris, 5 mM DTT, 10 mM reduced gluthatione pH 8.0 divided in four fractions of 50 μL. Control experiments were carried out loading only PSD-95 PDZ3 and hSR on the TALON^®^ and glutathione Sepharose^®^ resins, respectively. All experiments were performed in triplicate.

Protein quantification for bands at 12 kDa (PSD-95 PDZ3) in the TALON^®^ pull-down and at 38 kDa (hSR) for the glutathione Sepharose^®^ experiments was performed using a ChemiDoc gel imaging system (Bio-Rad, Hercules, CA, USA) considering as a reference the known protein amounts in the MW marker bands. The amount (µg) of PSD-95 PDZ3 in the band of the elution in the control experiment was subtracted to the amount of the same protein in the TALON^®^ pull-down to measure the effective amount of protein captured by the binding partner immobilized on the resin. The same procedure was performed for the analysis of hSR band in the elution for the glutathione Sepharose^®^ pull-down.

### 4.4. NMR Spectroscopy

The ^1^H-^15^N HSQC spectrum of an 80 μM sample of ^15^N-labeled PSD-95 PDZ-3 was acquired using a JEOL 600 MHz spectrometer. Unlabeled hSR (924 μM sample) was then added at increasing concentrations up to a 1:3 PDZ3:hSR molar ratio. All spectra were acquired at 25 °C in 50 mM Na phosphate buffer pH 7.8.

### 4.5. Cross-Linking

Cross-linking experiments were performed using 0.1% (*w*/*v*) glutaraldehyde as cross-linking agent. All cross-linking reactions were carried out in 50 mM HEPES pH 8.0, at room temperature, using 15 μM hSR and 30 μM PSD-95 PDZ3. The cross-linking reaction was stopped after 10 min with Tris HCl, pH 8.0 at a final concentration of 0.16 M. Control experiments were performed without glutaraldehyde or with glutaraldehyde and either hSR or PSD-95 PDZ3. Results of the cross-linking experiments were visualized by SDS-PAGE and analysed by ChemiDoc gel imaging system (Bio-Rad Laboratories, Hercules, CA, USA).

### 4.6. Isothermal Titration Calorimetry (ITC)

ITC titrations were performed at 25 °C using protein samples in a solution containing 50 mM HEPES, pH 8.0. All solutions were degassed for 10 min under vacuum before the titration. Experiments were carried out using a MicroCal PEAQ-ITC (Malvern, Malvern, UK). PSD-95 PDZ3 was added to the instrument measurement cell, containing 280 μL of hSR by a first addition of 0.4 μL and 18 subsequent additions of 2 μL. The concentrations of hSR and PSD-95 PDZ3 were 20 μM hSR and 600 μM PDZ3, respectively (different concentrations were tested, ranging from 20 μM to 30 μM for hSR in the cell, and from 300 μM to 800 μM for PDZ3 in the syringe, with no improvement of the signal). The time interval between the addition of each aliquot of titrant was 150 s. In order to obtain the dilution heat, the reaction cell was filled only with the buffer solution, while the syringe was filled with a 20 μM hSR solution in the same buffer. Experiments were performed under continuous stirring at 750 rpm. Binding isotherms were obtained by the integration of each injection peak followed by subtraction with blank experiment (dilution experiment). Titration curves were fitted using a single-site model by MicroCal PEAQ Analysis Software version 1.40 developed by Malvern (Malvern Instruments Ltd.: Microcal PEAQ-ITC Analysis Software, Malvern, UK).

### 4.7. Enzymatic Assays

The initial velocity of L-serine β-elimination catalyzed by hSR was monitored through a coupled assay with lactate dehydrogenase (LDH) that measures the pyruvate that spontaneously forms from α-aminoacrylate [16]. The LDH-coupled assay was carried out in a solution containing 50 mM HEPES, 150 mM NaCl, 5 mM DTT, 50 μM PLP, 2 mM MgCl_2_, 33 U/mL LDH and 320 μM NADH, pH 7.8 at 37 °C. hSR was added at a concentration of 0.44 μM in the presence and absence of 2 mM ATP. The reaction was triggered by the addition of 20 mM L-serine, and NADH consumption was followed at 340 nm using a Varian 4000 spectrophotometer. The enzymatic activity was evaluated in the presence and absence of PSD-95 PDZ3 at a final concentration of 500 μM. All reactions were carried out at 37 °C in triplicate. Control experiments were carried out by replacing PSD-95 PDZ3 with dialysis buffer from PSD-95 PDZ3 final equilibration.

## Supplementary Materials

The following supporting information can be downloaded at https://www.mdpi.com/article/10.3390/ijms23094959/s1. References [56,57] are cited in Supplementary Materials pdf.
