# Supplementary material for: Human Serine Racemase Weakly Binds the Third PDZ Domain of PSD-95
Source: Int J Mol Sci. 2022 Apr 29;23(9):4959. doi: 10.3390/ijms23094959 (PMC9105370; doi:10.3390/ijms23094959)
Supplement: Supplementary file 1 [file ijms-23-04959-s001.zip › ijms-1660271-supplementary.pdf]

Human serine racemase weakly binds the third PDZ domain of PSD-95

Roberta Giaccari[1], Francesco Marchesani[1], Carlotta Compari[1], Emilia Fisicaro[1], Andrea Mozzarelli[1,2], Barbara Campanini[1,3], Stefano Bettati[2,3,4], Stefano Bruno[1,3], Serena Faggiano[1,2*]

[1]Department of Food and Drug, University of Parma, 43124 Parma, Italy

[2]Institute of Biophysics, CNR, 56124 Pisa, Italy

[3]Biopharmanet TEC, University of Parma, 43124 Parma, Italy

[4]Department of Medicine and Surgery, University of Parma, 43124 Parma, Italy

*corresponding author. E-mail: serena.faggiano@unipr.it

SUPPLEMENTARY INFORMATION

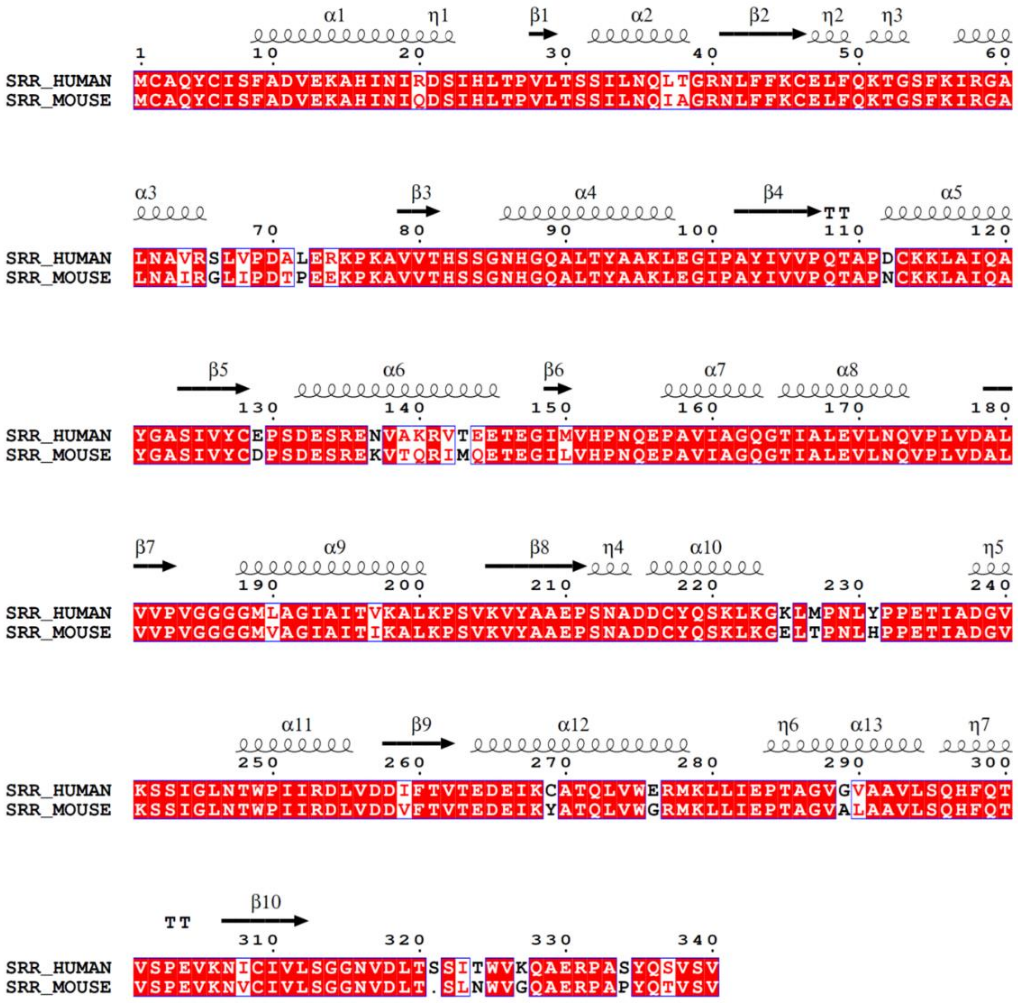

Figure S1: Amino acid sequence alignment of hSR (SSR_HUMAN) with mSR (SSR_MOUSE). The alignment was perfomed with the Align function in UniProt [1] and the similarity scores were calculated by the ESPript 3.0 progam [2]. Secondary structure elements above the alignment are derived from the hSR X-ray crystal structure, PDB code 5X2L.

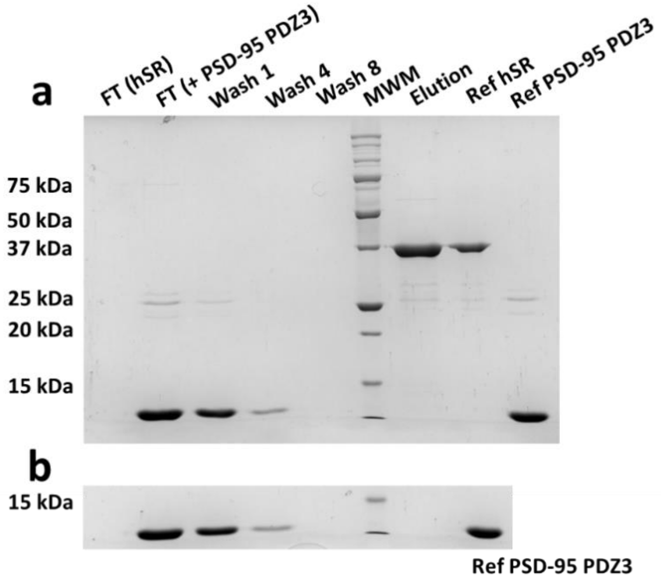

Figure S2: Pull down assay on TALON® resin with 400 µl wash. (**a**) Pull down assay on TALON® resin, immobilizing His-tagged hSR, with a wash volume of 400 µl divided in 8 steps of 50 µl each. FT is the flow-through after protein equilibration. Only Wash 1, 4 and 8 are reported on the gel. (**b**) Control experiment with only PSD-95 PDZ3 loaded onto the resin.

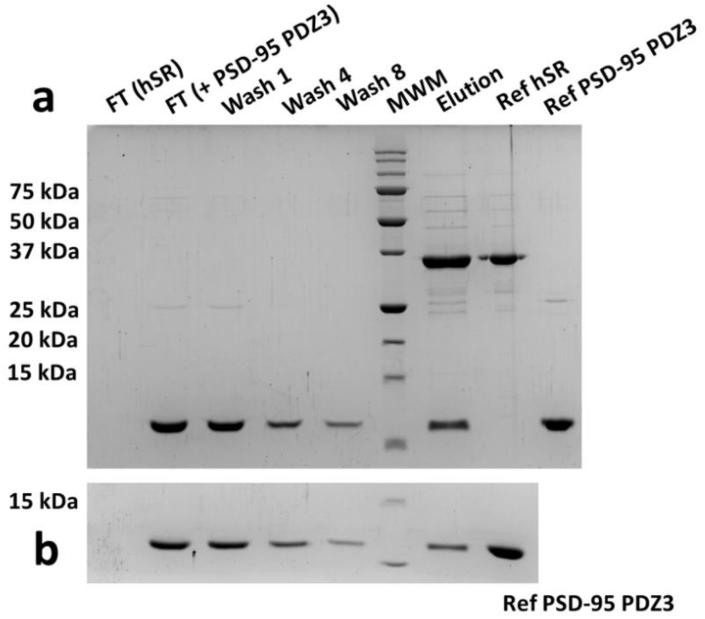

Figure S3: Pull down assay on Ni^{++} NTA resin. (**a**) Pull down assay on Ni^{++} NTA resin, immobilizing His-tagged hSR, with a wash volume of 400 µl divided in 8 steps of 50 µl each. FT is the flow-through after protein equilibration. Only Wash 1, 4 and 8 are reported on the gel. (**b**) Control experiment with only PSD-95 PDZ3 loaded onto the resin.

References:

1. The UniProt Consortium, UniProt: the universal protein knowledgebase in 2021. Nucleic Acids Research 2021, 49, (D1), D480-D489
2. Gouet, P., Courcelle, E., Stuart, D. I. & Metoz, F. ESPript: analysis of multiple sequence alignments in PostScript. Bioinformatics 1999, 15, 305-308.
